# 3D Bone Biomimetic Scaffolds for Basic and Translational Studies with Mesenchymal Stem Cells

**DOI:** 10.3390/ijms19103150

**Published:** 2018-10-13

**Authors:** Cristina Sobacchi, Marco Erreni, Dario Strina, Eleonora Palagano, Anna Villa, Ciro Menale

**Affiliations:** 1 CNR-IRGB, Milan Unit, via Fantoli 16/15, 20138 Milan, Italy; dario.strina@humanitasresearch.it (D.S.); eleonora.palagano@humanitasresearch.it (E.P.); anna.villa@humanitasresearch.it (A.V.); ciro.menale@humanitasresearch.it (C.M.); 2 Humanitas Research Hospital, via Manzoni 113, 20089 Rozzano, Italy; 3 Unit of Advanced Optical Microscopy, Humanitas Research Hospital, via Manzoni 113, 20089 Rozzano, Italy; marco.erreni@humanitasresearch.it

**Keywords:** mesenchymal stem cells, biomimetic scaffolds, 3D culture, regenerative medicine, soluble factor release

## Abstract

Mesenchymal stem cells (MSCs) are recognized as an attractive tool owing to their self-renewal and differentiation capacity, and their ability to secrete bioactive molecules and to regulate the behavior of neighboring cells within different tissues. Accumulating evidence demonstrates that cells prefer three-dimensional (3D) to 2D culture conditions, at least because the former are closer to their natural environment. Thus, for in vitro studies and in vivo utilization, great effort is being dedicated to the optimization of MSC 3D culture systems in view of achieving the intended performance. This implies understanding cell–biomaterial interactions and manipulating the physicochemical characteristics of biomimetic scaffolds to elicit a specific cell behavior. In the bone field, biomimetic scaffolds can be used as 3D structures, where MSCs can be seeded, expanded, and then implanted in vivo for bone repair or bioactive molecules release. Actually, the union of MSCs and biomaterial has been greatly improving the field of tissue regeneration. Here, we will provide some examples of recent advances in basic as well as translational research about MSC-seeded scaffold systems. Overall, the proliferation of tools for a range of applications witnesses a fruitful collaboration among different branches of the scientific community.

## 1. Introduction

Adult mesenchymal stem cells (MSCs) were first isolated from bone marrow and described as multipotent stromal cells with clonogenic capacity [1]. Afterwards, the possibility to isolate them from different tissues and to differentiate them towards diverse cell types fostered interest and research in the MSC field. In fact, many efforts have since been spent in order to elucidate their nature and properties and to develop cell-based therapies for a range of diseases [2,3,4,5]. In both contexts, the extracellular environment has been recognized as a critical factor influencing MSC behavior in terms of proliferation, survival, soluble factor release and differentiation [6]. In particular, biomaterials conquered the scene as versatile tools for the fabrication of three-dimensional (3D) structures for in vitro and in vivo applications [7,8,9].

In fact, in in vitro studies, 3D systems constitute a culture condition closer to native tissues as compared to a monolayer, may preserve and enhance cell functions and, overall, allow achievement of physiologically more relevant results [10,11]. Regarding in vivo applications specifically related to the skeletal compartment, since its origin, the field of bone tissue engineering has been feeling the need for replacement materials to fill tissue defects [12,13]. Synthetic polymers, ceramics, and metals, or tissue derivatives for auto/allografts, have been used (and still they are, in many instances), but these have a number of drawbacks and, in general, fail to recapitulate the quality of the original tissue [14]. Therefore, the aim scientists have been pursuing, which has been driving a huge development of materials science area, was to achieve autologous growth or tissue regeneration by means of a replacement material closely resembling the natural one, alone or in combination with MSCs [15]. Namely, they sought to identify optimal materials displaying biocompatibility (i.e., incorporation into host tissue without activation of an adverse immune response), biodegradability (i.e., limited persistence in the host), osteoconductivity, and osteoinductivity (i.e., recruitment of osteoprogenitor cells and differentiation induction and support), in order to reproduce bone extracellular environment and, therein, physiological cell behavior [8,16,17].

The bone microenvironment is complex in terms of composition, geometry, and mechanical properties. The extracellular matrix (ECM) comprises an organic part, mainly constituted by collagen and, in lower amount, non-collagenous proteins, such as proteoglycans, osteonectin, osteocalcin, and proteins of the SIBLING family; and an inorganic part, where calcium and phosphate are the most abundant ions, mainly in the form of hydroxyapatite (HA), and other ions, such as carbonate, citrate, magnesium, strontium, sodium, potassium, and fluoride, are present as trace elements [18]. In addition, many growth factors (GFs), such as FGF, TGFβ, BMPs, VEGF, and IGF I and II, are stored in the matrix and become available for cells in specific pathophysiological conditions [19]. 

The bone tissue displays a precise hierarchical organization [20]; tissue geometry and mechanical properties are strictly related and have been largely investigated on a macroscale, with respect to age, sex, specific disease status or medications, or lifestyle and fracture risk [21]. Extensive studies have been performed also at the cellular level, and the knowledge gained has inspired material choice and scaffold synthesis [22].

All these features influence cell fate and can be defined prior to material synthesis in order to trigger a specific cell behavior for the intended application [23,24]. Accordingly, a plethora of different biomaterials has been tested. The exploitation of the produced 3D scaffolds has allowed gaining insights into inherent MSC properties and designing diverse therapeutic strategies, taking advantage of tunable MSC multilineage differentiation capacity and secretory activity [25]. In the present minireview, we will describe most recent advances of basic and translational research about MSC-seeded scaffold systems specifically related to the bone tissue.

## 2. Use of 3D Scaffolds in Basic Studies: Evaluation of MSC Multilineage Potential and Study of Human Hematopoiesis

**Figure 1:**
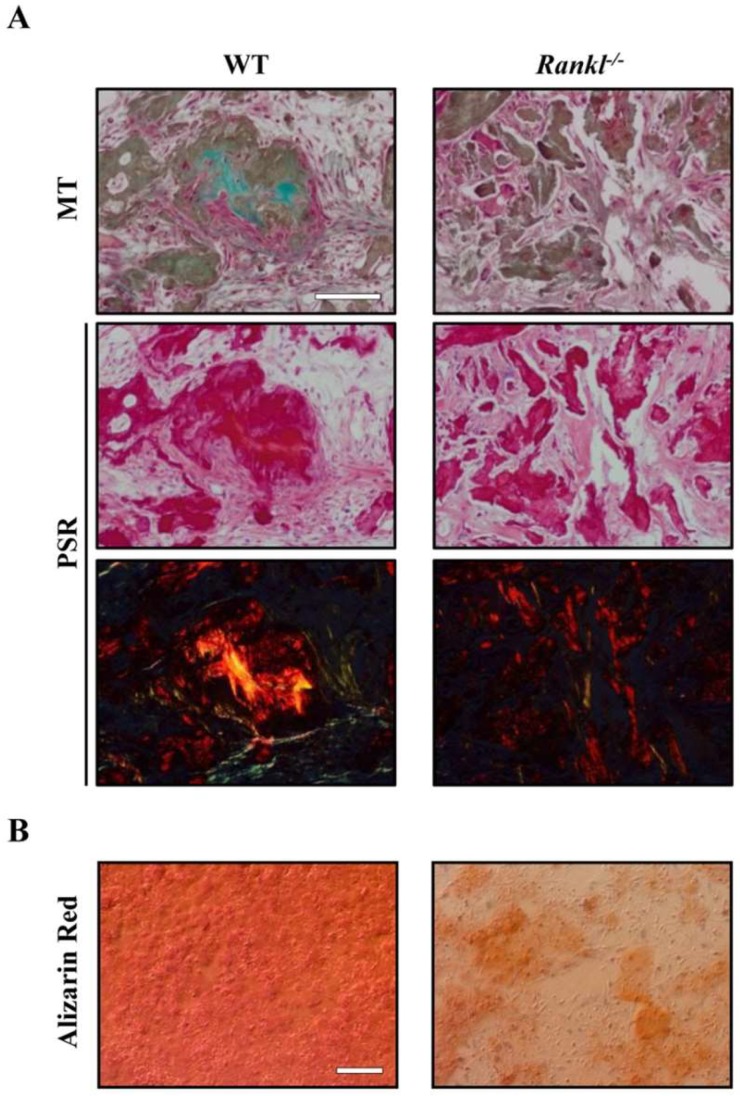
(**A**) Representative images of in vivo ceramic-based ectopic bone formation assay using wild type (WT) or *Rankl^−/−^* mesenchymal stem cells (MSCs). In scaffold systems seeded with WT MSCs, bone-like structures were present, as demonstrated by Masson’s trichrome (MT) staining of intense green collagen, and by yellow/orange birefringent fibers under polarized light in Picrosirius Red (PSR) staining. On the other hand, in *Rankl^−/−^* MSC-seeded scaffolds, the collagen deposition appeared less dense. Scale bar: 100 µm. (**B**) Representative images of in vitro WT or *Rankl^−/−^* MSC differentiation: MSCs were cultured in osteogenic medium for 14 days and mineralization was evaluated by Alizarin Red staining. Scale bar: 100 µm. Images are modified from [36].

A routine assay for MSC characterization is the evaluation of their capacity to differentiate into at least three different lineages (i.e., osteogenic, adipogenic, and chondrogenic) under specific stimuli in vitro. On the other hand, although not commonly performed, an even more relevant test is the assessment of their capacity to generate a histology-certified bone organ upon in vivo implantation [26]. Different protocols have been set up to this end [27,28,29,30]; the most frequently adopted one is based on MSC loading onto HA/tricalcium phosphate (HA/TCP) ceramic particles prior to implantation in immunocompromised mice, and harvesting of the formed ossicle about 2 months later [31,32,33,34]. Bioactive scaffolds represent a valuable alternative to ceramic particles, since they provide physical support and molecular cues for the seeded cells, which results in the activation of intracellular signaling promoting cell proliferation and differentiation [35]. In this regard, we recently performed this assay using murine bone marrow-derived wild type (WT) or *Rankl^−/−^* MSCs, and confirmed, in the latter, the partial osteogenic potential defect observed in vitro [36]. Briefly, for our purpose, we seeded 7 × 10^5^ WT or *Rankl^−/−^* MSCs onto a scaffold made of Mg-doped HA and type I collagen from equine tendon (MgHA/ColI), and implanted them subcutaneously into the back of immunocompromised NSG mice. Implants were harvested 2 months later. Histological analysis showed that all scaffolds were well colonized by cells and vascularized. Those bearing WT MSCs presented newly formed multifocal bone-like structures, as assessed by Masson’s trichrome and Picrosirius Red staining, as expected; on the other hand, those bearing *Rankl^−/−^* MSCs displayed areas of collagen deposition interspersed with fibroblasts/fibrocytes seeming more like a fibrous tissue, in line with in vitro results (Figure 1) [36]. 

Another important application of 3D scaffolds in basic studies is the investigation of the hematopoietic stem cell (HSC) niche, an extremely relevant topic whose direct examination in humans is prevented by obvious ethical issues and difficulties. Bourgine and colleagues produced an in vitro HSC niche using HA bone-like scaffolds seeded with human MSCs (hMSCs), taking advantage of a bioreactor system for cell culture in order to provide effective nutrients and oxygen supply and waste removal, and to support ECM synthesis [37]. The produced 3D stromal tissue was an adequate microenvironment for human CD34^+^ cell survival and expansion, and allowed preservation of their stemness features. Therefore, this MSC/scaffold-based 3D system was demonstrated to constitute a valuable technological platform for the study of human HSC biology in physiopathological conditions.

## 3. Effective Coupling of Cells and Scaffolds: The Material Choice for Better MSC Performance

Matrix components are input factors for the cells and affect their morphology, cytoskeletal organization, and integrin expression profile, as extensively investigated [38]. The aim to produce a physiologically relevant microenvironment for MSCs and, thereby, elicit appropriate responses for specific applications, has fueled the production of an overwhelming variety of scaffolding materials resembling bone ECM in terms of composition and properties, through chemical/physical modification processes [7,8,9,39]. A common strategy has been to combine synthetic polymers with HA in order to improve their bioactivity. For example, Guarino and colleagues incorporated magnesium and carbonate (MgCHA) particles into poly(ε-caprolactone) (PCL); this enhanced wettability of the composite surface, leading to significantly increased MSC adhesion, proliferation, in vitro mineralization, and in vivo bone formation [35]. In addition, natural polymers, including collagen, cellulose, chitosan, gelatin, alginate, and fibroin, have been exploited to develop scaffolds via biomimetic mineralization processes [40]. For example, Thompson and colleagues combined chondrogenically primed MSCs and hybrid composites made of collagen–hyaluronic acid or collagen/HA in a typical critical-sized bone defect assay [41]. They found higher new bone formation in the presence of the former biomaterial, and speculated this might be due to the increased VEGF secretion by the loaded MSCs, as assessed before implantation [41]. Other strategies used synthetic peptides with biological properties as building blocks for bioactive matrices, which offered the advantage to mimic both the ECM microarchitecture and chemistry [42]. Based on these considerations, Ramírez-Rodríguez and colleagues produced hybrid matrices made of recombinant type I collagen enriched with the RGD sequence (RCP), as cell attachment site, and defined the conditions for biomimetic mineralization [43]. In particular, they tested a scaffold mineralized in the presence of magnesium (MgApRCP) and found that this displayed low crystallinity, good permeability, homogeneous pore structure, and good interconnectivity, but lower porosity as compared to non-mineralized RCP, faster degradation, and ion release, likely favoring cell adhesion and osteogenic differentiation. MSC proliferation and osteogenic marker expression was higher on the MgApRCP scaffold. Cell migration was investigated, in detail, by longitudinally sectioning each sample, staining cell nuclei with DAPI, and analyzing three different levels by scanning electron microscopy. This demonstrated that MSCs migrated more in depth in the MgApRCP scaffold, which finely matched with the better performance of MSCs on this substrate for the other aspects evaluated [43]. 

An additional strategy is represented by coupling magnetic nanoparticles and scaffolds with stem cells, in the presence or absence of magnetic fields [44,45,46]. MSC seeding on magnetic scaffolds, and even more their exposure to magnetic fields, significantly increased cellular adhesion, osteogenic differentiation, angiogenesis, and bone regeneration, as compared to controls [44,45,46]. The underlying mechanisms consisted in activating signaling pathways such as those driven by integrins, BMP, MAPK, and NF-κB [47]. 

**Figure 2:**
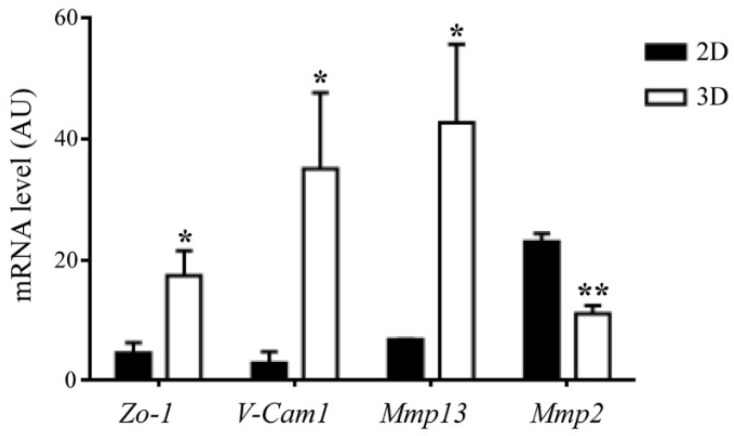
Gene expression analysis of the adhesion molecules *Zo-1* and *V-Cam1*, and of the metalloproteinases *Mmp13* and *Mmp2* in 2D- or 3D-cultured murine WT MSCs. Values are means ± SEM of 3–6 replicates. * *p* < 0.05, ** *p* < 0.005; evaluated by *t*-Test.

Finally, for what pertains our own experience, we carried out mouse MSC culture on the MgHA/ColI scaffold above mentioned, and investigated cell morphology and functions in this system by confocal microscopy, taking advantage of GFP expression by the cells and of collagen autofluorescence [48]. Briefly, previous reports had already proven that this kind of scaffold, produced through a biomimetic process in which HA nanoparticles nucleated directly on collagen fibers during their self-assembly, resembled “real” bone matrix. In fact, it was demonstrated to have low crystallinity and high wettability for optimal biocompatibility and cell adhesion, total porosity and pore size adequate for cell infiltration and nutrient diffusion, and degradation rate compatible with new bone formation rate [49]. The use of a specific collagen crosslinker influenced the mechanical behavior of the scaffold and the presence of Mg^2+^ benefited cellular activity [50], as could be expected based on the known importance of this trace element of the bone ECM for cellular activities and bone homeostasis [51]. Aiming to exploit these MgHA/ColI scaffolds in vivo in a very peculiar experimental setting (i.e., the *Rankl^−/−^* mouse, which displays severe growth retardation), the synthetic protocol was modified to scale down the scaffold size; despite these technical adaptations, physicochemical features capable of eliciting a specific cell fate for bioengineering applications were demonstrated [48]. In particular, as described, 24 h after MSC seeding, cells appeared to distribute on the upper surface of the scaffold, while as early as 48 h later, they were also found at greater depths. Moreover, at 48 and 72 h after seeding, the scaffold appeared to be modified and remodeled over time by seeded cells [48]. In fact, 3D-cultured MSCs modulated the expression of different metalloproteinases, with the upregulation of *Mmp13* and downregulation of *Mmp2*, compared to the 2D culture condition (Figure 2), which would also suggest a favored commitment towards the osteogenic cell fate in the 3D setting [52,53]. For a closer analysis, we seeded a lower number of cells (2.5 × 10^5^ cells) and, after overnight culture, we conducted confocal timelapse analysis, up to 24 h. In this way, we better visualized the MSCs’ typical fibroblast-like shape, and their interaction among each other and with collagen fibers (Video 1 in supplementary). Accordingly, gene expression analysis of 3D-cultured MSCs showed increased expression of adhesion molecules, such as *Zo-1* and *V-Cam1*, as compared to the 2D condition (Figure 2). 

Bone tissue regeneration proceeds through steps, comprising also osteoprogenitor recruitment, attachment, proliferation, and differentiation, therefore, scaffold design (and here, specifically, material choice) has to take into account all these cellular events and the sensitivity of cells to different stimuli [54,55]. In this framework, for example, Schofer and colleagues evaluated poly-l-lactic acid (PLLA) nanofibers functionalized with recombinant human BMP2, in a rat model of critical-sized cranial defect [56]. The authors showed that, after implantation these scaffolds were extensively colonized by cells expressing osteocalcin, bmp2, and smad5, which suggested activation of the osteoblast lineage. They also achieved early onset of bone regeneration, formation of bone marrow spaces, and osteointegration at the defect margins. Overall, PLLA/BMP2 nanofiber scaffolds, combining a supportive matrix for cell migration and an osteoinductive stimulus, demonstrated superior properties as compared to PLLA alone, which, in the same experimental model, led to limited bone formation, mainly in the marginal areas of the defect [56]. 

In the same line, since MSC recruitment is sensitive to the gradient of growth factors, cytokines, and other soluble factors, Aquino-Martinez and colleagues evaluated the effect of calcium release from a cell-free agarose/gelatin scaffold combined with different concentrations of CaSO_4_, in a murine calvarial bone defect model [57]. MSC migration and bone formation were proportional to CaSO_4_ concentration, and recruited progenitor cells showed activation of the PI3K/AKT pathway and increased osteoblast gene expression. Therefore, the authors proposed that scaffolds containing CaSO_4_ might be considered for applications in bone regeneration [57].

Furthermore, the production of apatite with features of “natural” bone in terms of sizes, specific surface area, carbonation degree, and surface composition, has been implemented [58]. Since the organic component of bone influences the mineralization process, nanoparticles and scaffolds have been made through biomineralization processes allowing nucleation of biomimetic HA nanoparticles on collagen fibers during their self-assembly, as also mentioned above [46,49,50,59]. Produced materials demonstrated high osteoinductivity and osteoconductivity and, of note, did reach the bedside [60].

Bone ECM itself has been employed after specific manipulations such as decellularization, demineralization, or deproteination [39]. The resulting modified ECM offered the advantage of displaying, simultaneously, many different chemical and physical instructive signals for the host cells, preserved osteoconductive and osteoinductive properties [61,62,63], and showed promising results also for potential exploitation in cartilage regeneration [64,65]. As our specific focus is on cell–biomaterial interaction, we will mention, as an example the work by Bourgine and colleagues, which tested the in vivo bone regeneration capacity of engineered and devitalized hypertrophic cartilage templates obtained in a two-step protocol: chondrogenic differentiation of hMSCs followed by genetically induced cell apoptosis [66]. This experimental strategy did not alter matrix protein composition, which was crucial for the in vivo performance of this material. In fact, in an ectopic implantation model, this cell-free hypertrophic cartilage ECM efficiently recruited the host cells and supported de novo bone tissue of host origin, including mature vasculature and a hematopoietic compartment. On the contrary, the same result was not obtained when devitalization was induced through freeze-and-thaw cycles, since these caused a significant loss of glycosaminoglycans, mineral content, and ECM-bound cytokines critically involved in inflammatory, vascularization, and remodeling processes. Overall, the authors demonstrated the capacity of customized ECM to activate endogenous regenerative programs by recapitulating tissue-specific developmental processes [66]. 

## 4. MSC-Scaffold Interaction: Mechanosensing and Mechanotransduction

MSCs can sense the ECM physical properties through engagement of membrane receptors, *in primis* integrins and focal adhesion proteins, and cytoskeletal modifications for “fast responses”, and activation of transcriptional programs for “slow responses”, in which the transcriptional regulators YAP/TAZ have a key role [67,68,69]. Through these mechanisms, MSCs translate the mechanical information into molecular signals leading to changes/adaptation in terms of cell morphology, adhesion, migration, and differentiation [9,70].

A number of studies investigated cell responses to material mechanical properties, including bulk and local stiffness, ligand density, and topography, in a 3D context, which is highly relevant to in vivo settings [71]. 

In recent years, Baker and colleagues developed a 3D fibrous synthetic material with light-tunable stiffness, functionalized with the cell-adhesive peptide RGD, and evaluated hMSC behavior on this substrate compared to an RGD-coupled flat hydrogel and a type I collagen matrix, for validation of their system [72]. They found that, in 3D systems, low fiber stiffness allowed cell recruitment of nearby fibers, as assessed by displacement of matrix-embedded fluorescent microspheres upon cell attachment. This increased ligand density at the cell surface and favored focal adhesions and downstream signaling, as assessed by vinculin localization and focal adhesion kinase phosphorylation; on the contrary, stiff fibers constrained cell spreading. Based on these results, the authors proposed that, in 3D, cell morphology was dictated more by fibrillar topography than by biochemical composition [72].

Yang and colleagues came to a similar conclusion by investigating the effect of spatial distribution, magnitude, and organization of the ECM mechanical properties on hMSC function [73]. In detail, they produced a hydrogel substrate containing a photodegradable crosslinker; light exposure of this material through a photomask allowed achievement of different percentages of stiff/soft areas organized in different ordered or randomized patterns. Cells seeded on hydrogels with orderly displayed stiff areas spread, elongated, and showed YAP activation proportional to the percentage of these areas. On the contrary, when stiff areas were distributed randomly in the material, actin organization was disrupted, and cells committed to a different fate [73].

Xie and colleagues investigated hMSC spreading, proliferation, migration, and differentiation in relation to fibrillar microarchitecture of a collagen gel whose physical parameters were determined by the polymerization temperature [74]. Namely, higher temperature led to thinner, more compact and less stiff fibers than lower temperature did and, in agreement with previous results, all the cell functions investigated were enhanced on this kind of fiber. Moreover, spreading MSCs were able to reshape the ECM through β1-integrin engagement, focal adhesion assembly, and myosin II-mediated contractility [74]. Interestingly, in this work, soft fibers favored osteogenic and stiff fibers’ adipogenic differentiation, at variance with other reports [67]. This discrepancy might be due to differences in experimental conditions and in the chemical composition of the substrates [67,68], and it likely reflects a yet incomplete understanding and mastery of MSC–biomaterial dynamics.

Sadowska and colleagues addressed the topic of cell–biomaterial interaction from an interesting point of view, which is the effect of ion exchange [75]. They underlined that a different composition and topography of the material could result in a different extent of ion exchange, which would affect cell behavior (particularly in static conditions), as cells uptake ions from the surrounding fluids. Specifically, these authors found that, in the presence of highly reactive substrates, a low ratio between the cell culture medium and the biomaterial caused lower cell adhesion, due to a reduced number of focal adhesions, cell shrinkage, reduced proliferation, and increased apoptosis [75]. Actually, these adverse effects could be prevented by exploiting bioreactor systems in which tuning of fluid flow could optimize soluble factor exchange [37,76,77]. Of course, all these observations are relevant in the set-up of bone regeneration strategies. 

Lastly, in recent years, 3D-miniaturized models have emerged also in the bone field, as useful tools mimicking organ-specific units in physiopathological conditions, in which what the biophysical stimuli cells are exposed to can be strictly controlled [78]. These systems offer the additional advantage of requiring a reduced amount of cells and reagents, thus allowing high-throughput evaluation of biological events [79]; in addition, they are often coupled with high-resolution imaging. Based on their properties, they have been used in basic studies, for example, to analyze the migratory behavior of hMSCs in the presence of different chemoattractants [80] and of osteoblasts following ECM degradation [81]; and also in translational studies, for drug development and testing [82]. They often require 3D bioprinting for the production of the 3D microtissue [83].

## 5. 3D Bioprinting for MSC Basic and Translational Studies

The implementation of 3D printing techniques has benefited basic and translational studies [84]. 3D printing is a powerful technology to fabricate, reproducibly and accurately, tissue-like structures with a geometry predefined by computer-aided designs, through layer-by-layer deposition of a cell-loaded biocompatible ink (bioink). Fine details can be included at a micrometer level, and highly complex structures can be synthetized [85,86]. Since the bioink is usually loaded with cells prior to printing and crosslinking, these are effectively embedded in the biomaterial. Some authors reported alterations of cell viability and functional properties due to shear stresses applied during the printing phase [87]; on the other hand, based on this concern, the bioink mechanical characteristics can be adapted in order to optimize cell survival and minimize impacts on cell phenotype and function [88]. Specifically aiming at favoring MSC osteogenic differentiation, points of reference of a 3D-printed scaffold comprise an interconnected porous structure to permit exchanges of nutrients, soluble factors, and other molecular signals; large pore size (1–3 mm) [89], high values of elastic modulus (up to 1600 MPa) and mechanical stability during scaffold degradation [90]. Different materials and microarchitectures have been assayed [91,92,93], and we will mention only a few, just to give a flavor of the flexibility of this technology and of the level of complexity that can be achieved. For example, Ferlin and colleagues demonstrated that an ordered interconnected cubic pore structure (1000 μm pore size, 80% porosity) within 3D-printed polymer resin scaffolds, either as such or modified with a mixture of collagen/HA or functionalized with TGFβ3, had a greater potential to support MSC differentiation as compared to a cylindrical one, likely due to higher tension [84]. Therefore, they proposed this system for single-step MSC enrichment and differentiation, potentially capable of avoiding phenotype changes which might occur following prolonged in vitro culture [84,94].

Holmes and colleagues designed very complicated structures to mimic the osteochondral region of articulate joints, a very challenging kind of tissue owing to its complex stratified architecture and diverse biomechanical properties [95]. These authors used a biocompatible polylactic acid polymer to print two different bi-phasic 3D models: the first displayed, on one side, a crosshatched pattern, reproducing the alignment of ECM and chondrocytes in the articulate cartilage and, on the other, an intersecting ring structure, representing the porous structure of subchondral bone. The latter model was named “bi-phasic key” and displayed the previous characteristics plus an internal structural feature traversing the length of the scaffold. Mechanical testing revealed better and shear compression properties in the key model, and surface modification with acetylated collagen, which is a typical component of the osteochondral ECM, enhanced MSC proliferation, and chondrogenic differentiation [95]. Based on their results, the authors proposed these biphasic scaffolds for application in personalized osteochondral regeneration and accordingly produced a prototype of a human knee. 

Zhou and colleagues described 3D-printed composite scaffolds made of photocurable polyethylene glycol diacrylate incorporating nanoHA and functionalized with the RGDS peptide to improve the substrate bioactivity, and tested 4 different pore geometries: large hexagonal (LH), small hexagonal (SH), large square (LS), and small square (SS) [96]. The produced scaffolds had different porosity (LH > (SH ~ LH) > SS), comparable wettability, and higher mechanical properties in the presence of higher porosity. MSC adhesion was higher on scaffolds with the SS pore geometry and low intensity pulsed ultrasound stimulation (LIPUS) further enhanced MSC proliferation, likely owing to mechanical stimulation of the adherent cells; in fact, LIPUS is already exploited in orthopedics as a biophysical stimulus to enhance musculoskeletal tissue repair [97].

Cunniffe and colleagues combined MSCs with a gene-activated bioink made of RGD-γ-irradiated alginate and nanoHA complexed to plasmid DNA expressing BMP2 and TGFβ3 [98]. Gene-activated bioinks have been proposed as an effective means to achieve localized sustained protein expression from transfected MSCs, leading to better performance compared to incorporation of GFs in the matrix, whence they may easily diffuse [99]. The authors used this bioink in 3D printing together with polycaprolactone, which provided mechanical stability to the construct. Gene-activated MSC-loaded constructs had high osteogenic potential in vitro and in vivo, after subcutaneous implantation in mice [98].

In order to pursue the same objective of prolonged GF retention within a 3D-printed scaffold, alginate sulfate has been exploited as a bioink with proven strong binding affinity for various GFs [100]. For example, Park and colleagues produced bioinks using alginate and alginate sulfate mixed with BMP2 and MC3T3-E1 osteoblasts prior to 3D printing, and evaluated the physicochemical properties of the two constructs and behavior of laden cells [101]. They found that both constructs had rheological properties suitable for 3D printing with different pore size and features, while alginate sulfate-containing constructs had a higher BMP2 retention, leading to higher osteoblast proliferation and differentiation in vitro [101]. The same strategy could be applied for the sustained delivery of other and/or multiple GFs and, thereby, be useful in the framework of different tissue engineering applications [101,102]. This kind of strategy has been undertaken in translational studies, for example, in the framework of articular cartilage regeneration. In this framework, Lee and colleagues produced a 3D-printed PCL meniscus scaffold seeded with poly(lactic-co-glycolic acid) (PLGA) microspheres encapsulating different growth factors between the meniscus inner and outer regions, which directed cell differentiation [103]. This strategy could be further improved by using templates with a specific collagen fiber architecture and orientation.

Moving closer to the bedside, considering the need for patient-specific graft substitutes for segmental bone repair, Chung and colleagues fabricated 3D-printed PLLA templates with a gradient in porosity and pore size resembling the native tissue, good biocompatibility under dynamic culture of hMSCs, and responsiveness to a physiologically relevant mechanical loading [104]. Therefore, this graft substitute was deemed of interest for exploitation in critical-sized bone defect correction in patients. 

Another interesting application of 3D-printed templates is in the field of craniofacial defects, where 3D printing showed the potential to achieve substantial aesthetic and functional improvements and will likely allow the implementation of personalized tools for rehabilitation, correction, and regeneration of these conditions [105,106].

## 6. MSC-Seeded Scaffolds as a Source of Soluble Factors 

A widely recognized and exploited property of MSCs is the release of soluble factors playing autocrine, paracrine, and systemic roles [107,108,109]. A recent study specifically investigated how scaffolds and their topographical features affect MSC paracrine function [110]. To this end, the authors employed PCL electrospun fibers with three different types of alignment: random (REF), aligned (AEF), and mesh (MEF). In addition to the influence on cell morphology exerted by the way of alignment and likely due to topography-related contact cues directing cell behavior (as reported also by others [9,70,111,112]), they observed an impact on MSC secretory profile. Namely, the expression and release of anti-inflammatory and wound healing-promoting factors, including COX2, PGE2, iNOS, TSG6, and TGFβ, and of the proangiogenic factors VEGF, HGF, and bFGF, which were increased in 3D-cultured MSCs as compared to cells cultured in a monolayer and, in particular, were higher in cells on AEF and MEF, as compared to those on REF [110]. This translated into a stronger induction of M2 macrophage polarization and endothelial cell proliferation and vessel-like formation in vitro, when RAW264.7 or HUVEC cells were treated with the conditioned medium from MSCs cultured on MEF and AEF structures [110]. Accordingly, we recently reported that murine *Rankl^−/−^* MSCs, transduced with a lentiviral vector expressing human soluble RANKL, secreted a larger amount of this cytokine when cultured on a bone biomimetic scaffold as compared to the 2D condition [48].

Moreover, MSC transduction and functionalization of biomaterials have been exploited in order to enhance soluble factor release and improve, or accelerate, specific processes. In general, matrix functionalization can be achieved through a variety of techniques and, based on this, soluble factor release can essentially occur through either diffusion-driven or erosion-driven mechanisms, following different kinetics [113,114]. Thus, this strategy adds further flexibility to cell-coupled (as well as cell-free) 3D scaffolds. For example, BMP2 overexpression, in MSCs as well as BMP2-scaffold conjugation, have been used in combination with different biomaterials, and led to higher osteogenic potential as compared to native MSCs, which could be relevant to accelerating bone regeneration [115,116]. Moreover, in the framework of a complex study design aiming at reproducing the osteoarticular junction, Stüdle and colleagues fabricated a bilayer cell-degradable poly(ethylene glycol) hydrogel which, in one layer, was functionalized with BMP2 or TGFβ3 and contained encapsulated hMSCs and, in the latter, contained nasal human nasal chondrocytes (NCs) [117]. TGFβ3 release induced in vitro chondrogenic differentiation and in vivo endochondral ossification from MSCs, and hypertrophic cartilage from NCs. On the other hand, when the hydrogel contained BMP2, a bone–cartilage composite was produced, in which stable hyaline cartilage was juxtaposed to the newly formed osteochondral tissue, thus demonstrating that this strategy could be further implemented, aiming to repair osteoarticular defects [117].

Other relevant GFs are, for example, VEGF and FGF9, because of their proangiogenic function that is particularly useful to enhance vessel formation and promote their stabilization and nutrient supply in the first phases of bone regeneration [19,118]. VEGF and FGF9 have been used either alone or in combination, evaluating different methods of GF conjugation to scaffold matrix, such as crosslinking into fibrin gels with Factor XIIIa [119].

Another class of molecules useful for conjugation with biomimetic materials is represented by miRNAs because of their known involvement in different diseases (for example, osteoporosis and osteoarthritis, for what pertains to bone) and of the wide availability of synthetic mimics or inhibitors [120,121]. Also, in this case, scaffold-based delivery systems, in which these small compounds are encapsulated or immobilized through different procedures, may result in sustained local concentration of the active molecule [122,123,124]. For example, Mencía Castaño and colleagues focused on miR16, which was demonstrated to have an inhibitory role on osteogenesis through downregulation of *Smad5*, *AcvR2a*, and *Runx2* [125]. Based on their previous experience in the field [126], the authors functionalized collagen-based scaffolds by soak-loading them with complexes of HA particles and anti-miR16, or blank HA particles as a control. Then, they seeded hMSCs dropwise on the scaffolds and, when mineralization was assessed, found a higher mineral calcium deposition in the presence of the specific antimiR [125]. Overall, this system appears versatile and suitable for diverse applications in bone tissue engineering. 

An alternative strategy is based on miRNAs or antimiR overexpression in MSCs. For example, based on data indicating a role for miR221 in the regulation of the osteogenic differentiation of human MSCs [127], Sadeghi and colleagues demonstrated that, in the model of a calvarial critical-sized defect, in situ implantation of PCL/HA nanofibers seeded with anti-miR221-transfected MSCs improved bone healing of the rat skull, thanks to increased vascularization and new bone formation [128]. From a translational point of view, targeting this miRNA could be of interest, with respect to a number of different skeletal pathological conditions in which it has been involved [129,130,131]. 

## 7. Conclusions

In conclusion, the development of 3D culture systems on engineered ECM has allowed for getting closer to the natural cell microenvironment and gaining deeper knowledge of MSC biology. Since essential “building blocks” have been identified, scientists have creatively matched them in many different ways in an ever-increasing variety of products. The few recent examples, here mentioned, give an idea of the enthusiasm and expectations pinned on this versatile technology; its possible complementation with computer-aided design makes it perfectly fit into the field of personalized medicine [95]. Moreover, similar strategies can be applied, also using either other kinds of pluripotent cells or differentiated cells, and this broadens their potential translational impact [132,133,134]. A number of clinical studies, in which tissue engineered grafts were exploited for bone regeneration, have been conducted, and promising results have been obtained [135], paving the way to a range of applications in MSC-based therapies, even though a long-term follow-up is not available, so far. Time will show whether results effectively meet expectations. 

In the meantime, there are some aspects requiring further improvement, such as the standardization and rigorous application of criteria for MSC characterization, methods for restraining immune system activation upon implantation, and sufficient scale-up for larger clinical trials. Continuation of the fruitful collaboration among diverse branches of the scientific community will likely achieve the goals.

## Supplementary Materials

Supplementary materials can be found at http://www.mdpi.com/1422-0067/19/10/3150/s1. **Video 1.** Cell-to-cell contact and alignment to collagen fibers assessed by timelapse confocal laser scanning microscopy of GFP-expressing mouse MSCs seeded on MgHA/ColI scaffold. Seeded cells were acquired for 8 h using a Leica SP8 Laser Scanning Confocal microscope, with a HC PLAN APO 10X/0.40 DRY objective. Emission filter bandwidths and sequential scanning acquisition were set up in order to avoid spectral overlap between fluorophores. GFP and collagen were excited with a 405 and 488 nm wavelength laser, respectively. The resulting fluorescence emission was collected in a wavelength range from 409 to 477 nm (for collagen) and from 494 to 650 nm (for GFP). Blue color corresponds to collagen fibers, green to MSCs. Scale bar: 50 μm.

## Abbreviations

2D	two-dimensional
3D	three-dimensional
AcvR2a	Activin A Receptor Type 2A
AEF	aligned electrospun fiber
AKT	Protein kinase B
bFGF	basic fibroblast growth factor
Bioink	biocompatible ink
BMP2	Bone Morphogenetic Protein 2
BMPs	Bone Morphogenetic Proteins
CaSO4	Calcium sulfate
COX2	cyclooxygenase-2
ECM	extracellular matrix
FGF	Fibroblast Growth Factor
FGF9	Fibroblast Growth Factor 9
GFP	Green Fluorescent Protein
GFs	Growth Factors
HA/TCP	HA/tricalcium phosphate
HA	Hydroxyapatite
HGF	Hepatocyte growth factor
hMSCs	human Mesenchymal Stem Cells
HSC	Hematopoietic Stem Cell
HUVEC	Human Umbilical Vein Endothelial Cells
IGF-I	Insulin-like Growth Factor-I
IGF-II	Insulin-like Growth Factor-II
iNOS	inducible Nitric Oxide Synthase
LH	Large Hexagonal
LIPUS	Low Intensity Pulsed Ultrasound Stimulation
LS	Large Square
MAPK	Mitogen-Activated Protein Kinase
MEF	Mesh Electrospun Fiber
Mg2+	Magnesium ion
MgApRCP	Mineralized, magnesium-doped recombinant type I collagen enriched with the RGD sequence
MgCHA	Magnesium and carbonate hydroxyapatite
MgHA/ColI	Magnesium-doped hydroxyapatite and type I collagen
miRNAs	microRNA
Mmp13	Matrix Metallopeptidase 13
Mmp2	Matrix Metallopeptidase 2
MPa	Megapascal
MSCs	Mesenchymal Stem Cells
MT	Masson’s Trichrome
NCs	nasal chondrocytes
NF-κB	nuclear factor of kappa light polypeptide gene enhancer in B cells
NSG	NOD scid gamma
PCL	poly(e-caprolactone)
PGE2	Prostaglandin E2
PI3K	Phosphoinositide 3 kinase
PLGA	poly-lactic-co-glycolic acid
PLLA	poly-L-lactic acid
PSR	PicroSirius Red
RANKL	Receptor activator of nuclear factor kappa-B ligand
RCP	Recombinant Collagen Peptide
REF	Random Electrospun Fiber
RGD	Arginine-Glycine-Aspartic acid
RGDS	Arginine-Glycine-Aspartic acid-Serine
Runx2	Runt-related transcription factor 2
SH	Small Hexagonal
Smad5	SMAD Family Member 5
SS	Small Square
TGFβ	Transforming Growth Factor beta
TGFβ3	Transforming Growth Factor beta-3
TSG6	TNF-Stimulated Gene 6 protein
V-Cam1	Vascular Cell adhesion molecule 1
VEGF	Vascular-Endothelial Growth Factor
WT	Wild Type
YAP/TAZ	Yes-associated protein/Tafazzin
Zo-1	Zonula occludens-1
