# Supplementary material for: 3D Bone Biomimetic Scaffolds for Basic and Translational Studies with Mesenchymal Stem Cells
Source: Int J Mol Sci. 2018 Oct 13;19(10):3150. doi: 10.3390/ijms19103150 (PMC6213614; doi:10.3390/ijms19103150)
Supplement: Supplementary file 1 [file ijms-19-03150-s001.zip › Supplementary Material.pdf]

**Acronyms used in the text and corresponding spelling in alphabetical order**

2D: two-dimensional

3D: three-dimensional

AcvR2a: Activin A Receptor Type 2A

AEF: aligned electrospun fiber

AKT: Protein kinase B

bFGF: basic fibroblast growth factor

Bioink: biocompatible ink

BMP2: Bone Morphogenetic Protein 2

BMPs: Bone Morphogenetic Proteins

$CaSO_4$: Calcium sulfate

COX2: cyclooxygenase-2

ECM: extracellular matrix

FGF: Fibroblast Growth Factor

FGF9: Fibroblast Growth Factor 9

GFP: Green Fluorescent Protein

GFs: Growth Factors

HA/TCP: HA/tricalcium phosphate

HA: hydroxyapatite

HGF: Hepatocyte growth factor

hMSCs: human Mesenchymal Stem Cells

HSC: Hematopoietic Stem Cell

HUVEC: Human Umbilical Vein Endothelial Cells

IGF-I: Insulin-like Growth Factor-I

IGF-II: Insulin-like Growth Factor-II

iNOS: inducible Nitric Oxide Synthase

LH: Large Hexagonal

LIPUS: Low Intensity Pulsed Ultrasound Stimulation

LS: Large Square

MAPK: Mitogen-Activated Protein Kinase

MEF: Mesh Electrospun Fiber

$Mg^{2+}$: Magnesium ion

MgApRCP: mineralized, magnesium-doped recombinant type I collagen enriched with the RGD sequence

MgCHA: magnesium and carbonate hydroxyapatite

MgHA/ColI: Magnesium-doped hydroxyapatite and type I collagen

miRNAs: microRNA

Mmp13: Matrix Metallopeptidase 13

Mmp2: Matrix Metallopeptidase 2

MPa: Megapascal

MSCs: Mesenchymal Stem Cells

MT: Masson's Trichrome

NCs: nasal chondrocytes

NF-κB: nuclear factor of kappa light polypeptide gene enhancer in B cells

NSG: NOD scid gamma

PCL: poly(ε-caprolactone)

PGE2: Prostaglandin E2

PI3K: Phosphoinositide 3 kinase

PLGA: poly-lactic-co-glycolic acid

PLLA: poly-L-lactic acid

PSR: PicroSirius Red

RANKL: Receptor activator of nuclear factor kappa-B ligand

RCP: Recombinant Collagen Peptide

REF: Random Electrospun Fiber

RGD: Arginine-Glycine-Aspartic acid

RGDS: Arginine-Glycine-Aspartic acid-Serine

Runx2: Runt-related transcription factor 2

SH: Small Hexagonal

Smad5: SMAD Family Member 5

SS: Small Square

TGFβ: Transforming Growth Factor beta

TGFβ3: Transforming Growth Factor beta-3

TSG6: TNF-Stimulated Gene 6 protein

V-Cam1: Vascular Cell adhesion molecule 1

VEGF: Vascular-Endothelial Growth Factor

WT: Wild Type

YAP/TAZ: Yes-associated protein/Tafazzin

Zo-1: Zonula occludens-1
